# Analysis of solitary wave solutions in the fractional-order Kundu–Eckhaus system

**DOI:** 10.1038/s41598-024-53330-7

**Published:** 2024-02-14

**Authors:** Saleh Alshammari, Khaled Moaddy, Rasool Shah, Mohammad Alshammari, Zainab Alsheekhhussain, M. Mossa Al-sawalha, Mohammad Yar

**Affiliations:** 1 Department of Mathematics, College of Science, University of Hail, 2440 Hail, Saudi Arabia; 2 Department of Mathematics, College of Science and Humanities, Shaqra University, 11691 Shaqra, Saudi Arabia; 3 Department of Computer Science and Mathematics, Lebanese American University, Beirut, Lebanon; 4 Department of Mathematics, Kabul Polytechnic University, Kabul, Afghanistan

**Keywords:** Mathematics and computing, Optics and photonics

## Abstract

The area of fractional partial differential equations has recently become prominent for its ability to accurately simulate complex physical events. The search for traveling wave solutions for fractional partial differential equations is a difficult task, which has led to the creation of numerous mathematical approaches to tackle this problem. The primary objective of this research work is to provide optical soliton solutions for the Frictional Kundu–Eckhaus equation (FKEe) by utilizing generalized coefficients. This strategy utilizes the Riccati–Bernoulli sub-ODE technique to effectively discover the most favorable traveling wave solutions for fractional partial differential equations. As a result, it facilitates the extraction of optical solitons and intricate wave solutions. The Backlund transformation is used to methodically construct a sequence of solutions for the specified equations. The study additionally showcases 3D and Density graphics that visually depict chosen solutions for certain parameter selections, hence improving the understanding of the outcomes.

## Introduction

The study of nonlinear wave equations is a widespread and essential endeavor in the domain of applied mathematics and theoretical physics, with implications for diverse domains such as engineering and the biological sciences. These equations provide valuable models for dynamic systems characterized by a diverse range of phenomena. Some examples of these phenomena are solitary waves, rogue waves, the appearance of singularities, dispersive turbulence, and the spread of chaos. Nonlinear wave phenomena occur in various physical and natural systems and have important applications in fields including nonlinear optics, fiber optics, the study of water and atmospheric waves, and the analysis of turbulence in hydrodynamics and plasmas. Examining these nonlinear wave equations provides vital insights into the behavior of complex systems across various scientific fields, facilitating advancements and discoveries in both theoretical and practical applications^1–4^.

Fractional calculus, an expansion of traditional calculus, has a significant impact on accurately representing the complexities of the physical world in comparison to integer-order^5,6^ partial differential equations. The field of fractional-order nonlinear partial differential equations comprises a range of innovative interpretations of traditional integer-order differential equations, using widely accepted formulations such as Riemann-Liouville and Caputo fractional derivatives. In recent decades, there has been significant interest in studying fractional-order nonlinear partial differential equations due to their wide range of applications in areas such as viscoelasticity, dielectric polarization, electrode-electrolyte polarization, electromagnetic wave propagation, quantitative finance, and the quantum evolution of complex systems^7^. The fractional-order nonlinear partial differential equations are more suitable models for a wide range of real-world issues, surpassing the effectiveness of their integer-order counterparts. Recent academic research has examined different aspects of fractional-order nonlinear partial differential equations, including proving the existence and uniqueness of solutions for Cauchy-type fractional equations, creating explicit and numerical solution methods, and evaluating the stability of solutions^8,9^. Moreover, significant focus has been dedicated to the pursuit of precise solutions for non-linear partial differential equations that govern essential physical and dynamic phenomena.

A wide range of techniques have been utilized in scientific literature to tackle fractional differential equations^10,11^. These techniques include, but are not limited to, the Laplace transformation method, Fourier transformation method, iteration methods, and operational methods^12^. Each of these methods provides a distinct approach to solving these intricate mathematical problems.These strategies are mostly suitable for solving particular types of linear fractional differential equations that have constant coefficients. Nevertheless, a specific group of research projects has adopted an innovative strategy, utilizing strong and effective techniques to calculate both numerical and precise solutions for nonlinear partial differential equations with fractional orders. Some notable approaches in this field are the Adomian decomposition method^13–15^, the variational iterative method^16,17^, the homotopy perturbation method^18,19^, the fractional Riccati sub-equation method^20,21^, and the spectral Tau method^22^. It is crucial to emphasize that in this research, Jumarie et al. have successfully determined precise and numerical solutions for fractional-order nonlinear partial differential equations, notably by utilizing the concept of the modified Riemann-Liouville derivative^23^. These novel methods have made it easier to study a wide range of fractional-order nonlinear partial differential equations, expanding our knowledge in this field. In addition, El-Sayed^13,14^ et. al have perceptively recognized the difficulties involved in determining precise solutions for fractional-order nonlinear partial differential equations. They stressed the importance of developing an efficient and intuitive approach specifically designed for these equations, acknowledging that finding a universal solution for all forms of nonlinear fractional issues is still challenging. An impressive addition to this area of study is the robust and effective technique introduced by Choi et al.^24^, which provides a convincing strategy for obtaining precise solutions to fractional-order nonlinear partial differential equations, expanding the range of instruments accessible to address these complex mathematical problems.

This work specifically aims to provide accurate optical soliton wave solutions for fractional-order Kundu–Eckhaus equation (FKEe). In order to achieve this, we utilize the Riccati–Bernoulli sub-ODE technique. By using this method in combination with Backlund transformation, we obtain precise solutions for the specified non-linear partial differential equations with fractional orders. These solutions provide useful insights into the behavior of wave events within the given equations. In the conclusion part of the paper, we summarize our findings and highlight the main insights gained from our investigation of these fractional-order nonlinear ppartial differential equations, which enhance our comprehension of the dynamics and features of these complex mathematical models. This study aims to investigate the Frictional Kundu–Eckhuas equation (FKEe), which may be expressed as:

1 $$\begin{aligned} \begin{aligned} \iota \,D_t^\alpha {q}+lD_x^\beta \big (D_x^\beta {q}\big )+z|{q}|^4{q}+{h}D_x^\beta \big (|{q}|^2\big ){q}=0, 0<\alpha ,\beta \le 1. \end{aligned} \end{aligned}$$

We have two independent variables in the given equation, *x*, which stands for the spatial variable, and *t* which stands for the temporal variable. The soliton pulse profile is correlated with the dependent variable *q*(*x*, *t*). The first term in Eq. (1) controls the nonlinear wave evolution. Different physical qualities are connected with the real-valued constants (*l*), (*z*) and (h), representing, group velocity dispersion, quintic nonlinearity, and nonlinear dispersion respectively. The fractional derivatives used in Eq. (1) are in accordance with conformable fractional derivatives. The operator denoting these derivatives of order $\delta$ is defined in accordance with the notation given in reference^25^ as follows:

2 $$\begin{aligned} \begin{aligned} D_\theta ^\delta {q(\theta )}=\lim _{m \rightarrow 0} \frac{q(m(\theta )^{1-\delta }-q{(\theta )})}{m},0<\delta \le 1. \end{aligned} \end{aligned}$$

This inquiry utilizes the following characteristics of this derivative:

3 $$\begin{aligned}{} & {} D_\theta ^\delta \theta ^j=j \theta ^{j-\delta }. \end{aligned}$$

4 $$\begin{aligned}{} & {} D_\theta ^\delta \left( j_1 \eta (\theta ) \pm j_2 m(\theta )\right) =j_1 D_\theta ^\delta (\eta (\theta )) \pm j_2 D_\theta ^\delta (m(\theta )). \end{aligned}$$

5 $$\begin{aligned}{} & {} D_\theta ^\delta \chi \left[ \xi ^\tau (\theta )\right] =\chi _{\xi }^{\prime }(\xi (\theta )) D_\theta ^\delta \xi (\theta ). \end{aligned}$$

## Methodology

Consider the scenario in which we are confronted with the subsequent nonlinear frictional partial differential equation:

6 $$\begin{aligned} F\left( f, \partial ^\alpha _t (f),\partial ^\beta _\zeta (f), \partial ^{2\alpha }_t (f),\partial ^{2\beta }_\zeta (f),f^2, \ldots \right) = 0, 0<\alpha ,\beta \le 1. \end{aligned}$$

The polynomial *F* is one that depends on the function $f(\zeta ,t)$ and its frictional derivatives. The nonlinear terms as well as the frictional order derivatives are included in this polynomial. We next go over the main steps of this technique in detail.

In order to investigate potential solutions for Eq. (1), we propose the subsequent complex wave transformations:

7 $$\begin{aligned}{} & {} F=q(x,t)=f(\zeta )e^{\iota \vartheta (x,t)}. \end{aligned}$$

8 $$\begin{aligned}{} & {} \zeta =k\left( \frac{x^\beta }{\beta }-v\frac{t^\alpha }{\alpha }\right) . \end{aligned}$$

9 $$\begin{aligned}{} & {} \vartheta (x,t)=-m\frac{x^\beta }{\beta }+\omega \frac{t^\alpha }{\alpha }+\theta . \end{aligned}$$

The symbol (*k*) denotes the gradient of the connection between the two stable states within the framework of a soliton solution. Furthermore, this solution is distinguished by specific parameters like soliton velocity, frequency, wave number, and the phase constant of the soliton, represented by $(v), (m), (\omega )$ and $(\theta$). Equation (1) can be transformed into the following ode by applying transformation.

10 $$\begin{aligned} F\left( f, f'(\zeta ),f''(\zeta ), f'''(\zeta ), \ldots \right) = 0. \end{aligned}$$

Consider the formal solution for Eq. (10)

11 $$\begin{aligned} {f}(\zeta )=\sum _{i=-n}^{n} c_i{\psi (\zeta )}^i. \end{aligned}$$

The $c_i$ constants must be found under the constraint that $c_n \ne 0$, $c_{-n} \ne 0$. Meanwhile, the Backlund transformation that follows produces the function.

12 $$\begin{aligned} \psi (\zeta ) = \frac{-\kappa {\mathcal {B}} + {\mathcal {A}}\phi (\zeta )}{{\mathcal {A}} + {\mathcal {B}}\phi (\zeta )}. \end{aligned}$$

Let $\kappa$, ${\mathcal {A}}$, and ${\mathcal {B}}$ be constants, with the condition that ${\mathcal {B}}\ne 0$. Additionally, let $\phi (\zeta )$ be a function that may be defined as:

13 $$\begin{aligned} \frac{d{\phi }}{d\zeta }=\kappa +{\phi (\zeta )^2}. \end{aligned}$$

The solutions of Eq. (13) are widely recognised^26^ to be as follows:

(i) If $\kappa <0$, then $\phi (\zeta )=-\sqrt{-\kappa }\tanh (\sqrt{-\kappa }\zeta )$, or $\phi (\zeta )=-\sqrt{-\kappa }coth(\sqrt{-\kappa }\zeta )$.
(ii) If $\kappa >0$, then $\phi (\zeta )=\sqrt{\kappa }\tan (\sqrt{\kappa }\zeta )$, or $\phi (\zeta )=-\sqrt{\kappa }cot(\sqrt{\kappa }\zeta )$.
(iii) If $\kappa =0$, then $\phi (\zeta )=\frac{-1}{\zeta }$.

Applying homogeneous balancing principles, which involve striking a balance between the nonlinear variables in Eq. (11) and the highest order derivatives, allows for the determination of the positive integer (*N*) within the context of Eq. (12). To be more precise, we can write ${f}(\zeta )$ degree as $D[{f}(\zeta )]=N$. This therefore allows us to calculate the degree of related expressions in the following way:

14 $$\begin{aligned} D\left[ \frac{d^p f}{d\zeta ^p}\right] = N + p, \quad D\left[ f^J \frac{d^p f}{d\zeta ^p}\right] ^s = NJ + s(p + N). \end{aligned}$$

By combining Eqs. (11) and (13) with Eq. (10), and then grouping together terms that have the same powers of ${f(\zeta )}$ and equating them to zero, we establish a set of algebraic equations. The efficient resolution of this system can be achieved by applying Maple software to infer the relevant values for $(c_i)$, (*l*), (*h*), (*b*), (*z*), (*k*) and (*v*). Consequently, this facilitates the accurate calculation of the solutions for Eq. (6) that propagate as soliton waves, achieved by computational analysis.

## Mathematical formulation

In this section, we will utilize the method described in Section 2 to determine the exact solitary wave solutions for the Frictional Kundu–Eckhaus model (1). To achieve this goal, we utilize the wave transformation outlined in Eq. (7), in order to streamline Eq. (1) into real and imaginary parts.

15 $$\begin{aligned}{} & {} lk^2F''(\zeta )-(v+lk^2)F(\zeta )+2hkF^2(\zeta )F'(\zeta )+zF^5(\zeta )=0. \end{aligned}$$

16 $$\begin{aligned}{} & {} v=-2lk. \end{aligned}$$

The differential equation $f''$ and $f^5$ can be balanced by setting the parameter, $N=\frac{1}{2}$. In order to obtain analytical solutions, a transformation is utilized.

17 $$\begin{aligned} F=f^{\frac{1}{2}}. \end{aligned}$$

Upon substitution this transformation into Eq. (15), the resulting expression is produced.

18 $$\begin{aligned} lk^2f''(\zeta )f(\zeta )+4hkf^2(\zeta )f'(\zeta )-lk^2(f'(\zeta ))^2-4(v+lk^2)f^2(\zeta )+4zf^5(\zeta )=0. \end{aligned}$$

Equation (18) denotes the nonlinear ordinary differential equation form of Eq. (1). By applying the homogeneous balance principle, we can determine that the balancing constant, denoted as $N=1$ is obtained.

In the present study, we utilize the substitution of Eq. (11) in combination with Eqs. (13) and (10) to incorporate them into Eq. (18). A system of algebraic equations is formulated by carefully collecting the coefficients associated with $\psi ^{i}(\zeta )$, and afterwards equating them to zero. By utilizing Maple software as a computational tool, we effectively solve the above system of algebraic equations, resulting in the succeeding outcomes:

### Case 1

19 $$\begin{aligned} \begin{aligned}{}&c_{{1}}=c_{{1}},c_{{-1}}=-c_{{1}}\kappa ,c_{{0}}=0,v=4\,hkc_{{1}} \kappa ,l=0,h=h,{\mathcal {B}}={\mathcal {B}},z=-{ \frac{hk}{c_{{1}}}},k=k. \end{aligned} \end{aligned}$$

### Case 2

20 $$\begin{aligned} \begin{aligned}{}&c_{{1}}=c_{{1}},c_{{-1}}=c_{{1}}\kappa ,c_{{0}}=0,v=- \left( \kappa +1 \right) l{k}^{2},l=l,h=0,{\mathcal {B}}={\mathcal {B}},z=-3/4\,{\frac{{k}^{2}l}{{c_{{1}}}^{2}}},k=k. \end{aligned} \end{aligned}$$

### Case 3

21 $$\begin{aligned} \begin{aligned}{}&c_{{1}}=1/2\,\sqrt{-{\kappa }^{-1}}c_{{0}},c_{{-1}}=-1/2\,\sqrt{-{ \kappa }^{-1}}c_{{0}}\kappa ,c_{{0}}=c_{{0}},v=- \left( 4\,\kappa +1 \right) l{k}^{2},l=l,h=-2\,kl{\frac{1}{\sqrt{-{\kappa }^{-1}}}}{c_{{0 }}}^{-1},\\&{\mathcal {B}}={\mathcal {B}},z=-{\frac{l{ k}^{2}\kappa }{{c_{{0}}}^{2}}},k=k. \end{aligned} \end{aligned}$$

Assuming case 1, we get the following families of solutions:

### Family 1

When $\kappa <0$ then Eq. (1) have the following solitary wave solutions:

22 $$\begin{aligned} \begin{aligned} F_1(x,t)&=-c_{{1}}\sqrt{-\kappa }\tanh \left( \sqrt{-\kappa }k \left( {\frac{{x }^{\beta }}{\beta }}-4\,{\frac{hkc_{{1}}\kappa \,{t}^{\alpha }}{\alpha }} \right) \right) {e}^{i \left( -{\frac{m{x}^{\beta }}{\beta }}+{\frac{\omega \,{t}^{\alpha }}{\alpha }}+\theta \right) }+c_{{1}}\kappa \,{e}^{i \left( -{\frac{m{x}^{\beta }}{\beta }}+{\frac{\omega \,{t}^{\alpha }}{ \alpha }}+\theta \right) }\\&\quad \times {\frac{1}{\sqrt{-\kappa }}} \left( \tanh \left( \sqrt{-\kappa }k \left( {\frac{{x}^{\beta }}{\beta }}-4\,{ \frac{hkc_{{1}}\kappa \,{t}^{\alpha }}{\alpha }} \right) \right) \right) ^{-1}. \end{aligned} \end{aligned}$$

or

23 $$\begin{aligned} \begin{aligned} F_2(x,t)&= -c_{{1}}\sqrt{-\kappa }\coth \left( \sqrt{-\kappa }k \left( {\frac{{x }^{\beta }}{\beta }}-4\,{\frac{hkc_{{1}}\kappa \,{t}^{\alpha }}{\alpha }} \right) \right) {e}^{i \left( -{\frac{m{x}^{\beta }}{\beta }}+{\frac{\omega \,{t}^{\alpha }}{\alpha }}+\theta \right) }+c_{{1}}\kappa \,{e}^{i \left( -{\frac{m{x}^{\beta }}{\beta }}+{\frac{\omega \,{t}^{\alpha }}{ \alpha }}+\theta \right) }\\&\quad \times {\frac{1}{\sqrt{-\kappa }}} \left( \coth \left( \sqrt{-\kappa }k \left( {\frac{{x}^{\beta }}{\beta }}-4\,{ \frac{hkc_{{1}}\kappa \,{t}^{\alpha }}{\alpha }} \right) \right) \right) ^{-1}. \end{aligned} \end{aligned}$$

### Family 2

When $\kappa >0$ then Eq. (1) have the following solitary wave solutions:

24 $$\begin{aligned} \begin{aligned} F_3(x,t)&=c_{{1}}\sqrt{\kappa }\tan \left( \sqrt{\kappa }k \left( {\frac{{x}^{ \beta }}{\beta }}-4\,{\frac{hkc_{{1}}\kappa \,{t}^{\alpha }}{\alpha }} \right) \right) {e}^{i \left( -{\frac{m{x}^{\beta }}{\beta }}+{\frac{\omega \,{t}^{\alpha }}{\alpha }}+\theta \right) }-c_{{1}}\sqrt{\kappa } {e}^{i \left( -{\frac{m{x}^{\beta }}{\beta }}+{\frac{\omega \,{t}^{ \alpha }}{\alpha }}+\theta \right) }\\&\quad \times \left( \tan \left( \sqrt{\kappa }k \left( {\frac{{x}^{\beta }}{\beta }}-4\,{\frac{hkc_{{1}}\kappa \,{t}^{ \alpha }}{\alpha }} \right) \right) \right) ^{-1}. \end{aligned} \end{aligned}$$

or

25 $$\begin{aligned} \begin{aligned} F_4(x,t)&=-c_{{1}}\sqrt{\kappa }\cot \left( \sqrt{\kappa }k \left( {\frac{{x}^{ \beta }}{\beta }}-4\,{\frac{hkc_{{1}}\kappa \,{t}^{\alpha }}{\alpha }} \right) \right) {e}^{i \left( -{\frac{m{x}^{\beta }}{\beta }}+{\frac{\omega \,{t}^{\alpha }}{\alpha }}+\theta \right) }+c_{{1}}\sqrt{\kappa } {e}^{i \left( -{\frac{m{x}^{\beta }}{\beta }}+{\frac{\omega \,{t}^{ \alpha }}{\alpha }}+\theta \right) }\\&\quad \times \left( \cot \left( \sqrt{\kappa }k \left( {\frac{{x}^{\beta }}{\beta }}-4\,{\frac{hkc_{{1}}\kappa \,{t}^{ \alpha }}{\alpha }} \right) \right) \right) ^{-1}. \end{aligned} \end{aligned}$$

### Family 3

 When $\kappa =0$, then Eq. (1) have the following solitary wave solutions:

26 $$\begin{aligned} \begin{aligned} F_5(x,t)&=-c_{{1}}{e}^{i \left( -{\frac{m{x}^{\beta }}{\beta }}+{\frac{\omega \,{ t}^{\alpha }}{\alpha }}+\theta \right) }{k}^{-1} \left( {\frac{{x}^{ \beta }}{\beta }}-4\,{\frac{hkc_{{1}}\kappa \,{t}^{\alpha }}{\alpha }} \right) ^{-1}\\&\quad +c_{{1}}\kappa \,k \left( {\frac{{x}^{\beta }}{\beta }}-4 \,{\frac{hkc_{{1}}\kappa \,{t}^{\alpha }}{\alpha }} \right) {e}^{i \left( -{\frac{m{x}^{\beta }}{\beta }}+{\frac{\omega \,{t}^{\alpha }}{ \alpha }}+\theta \right) }. \end{aligned} \end{aligned}$$

Assuming case 2, we get the following families of solutions:

### Family 4

: When $\kappa <0$ then Eq. (1) have the following solitary wave solutions:

27 $$\begin{aligned} \begin{aligned} F_6(x,t)&=c_{{1}}\sqrt{-\kappa }\tanh \left( \sqrt{-\kappa }k \left( {\frac{{x} ^{\beta }}{\beta }}+{\frac{ \left( \kappa +1 \right) l{k}^{2}{t}^{\alpha }}{\alpha }} \right) \right) {e}^{i \left( -{\frac{m{x}^{\beta }}{ \beta }}+{\frac{\omega \,{t}^{\alpha }}{\alpha }}+\theta \right) }+c_{{1} }\kappa \,{e}^{i \left( -{\frac{m{x}^{\beta }}{\beta }}+{\frac{\omega \, {t}^{\alpha }}{\alpha }}+\theta \right) }{\frac{1}{\sqrt{-\kappa }}}\\&\quad \times \left( \tanh \left( \sqrt{-\kappa }k \left( {\frac{{x}^{\beta }}{ \beta }}+{\frac{ \left( \kappa +1 \right) l{k}^{2}{t}^{\alpha }}{\alpha } } \right) \right) \right) ^{-1}. \end{aligned} \end{aligned}$$

or

28 $$\begin{aligned} \begin{aligned} F_7(x,t)&=c_{{1}}\sqrt{-\kappa }\coth \left( \sqrt{-\kappa }k \left( {\frac{{x} ^{\beta }}{\beta }}+{\frac{ \left( \kappa +1 \right) l{k}^{2}{t}^{\alpha }}{\alpha }} \right) \right) {e}^{i \left( -{\frac{m{x}^{\beta }}{ \beta }}+{\frac{\omega \,{t}^{\alpha }}{\alpha }}+\theta \right) }+c_{{1} }\kappa \,{e}^{i \left( -{\frac{m{x}^{\beta }}{\beta }}+{\frac{\omega \, {t}^{\alpha }}{\alpha }}+\theta \right) }{\frac{1}{\sqrt{-\kappa }}}\\&\quad \times \left( \coth \left( \sqrt{-\kappa }k \left( {\frac{{x}^{\beta }}{ \beta }}+{\frac{ \left( \kappa +1 \right) l{k}^{2}{t}^{\alpha }}{\alpha } } \right) \right) \right) ^{-1}. \end{aligned} \end{aligned}$$

### Family 5

 When $\kappa >0$ then Eq. (1) have the following solitary wave solutions:

29 $$\begin{aligned} \begin{aligned} F_8(x,t)&=-c_{{1}}\sqrt{\kappa }\tan \left( \sqrt{\kappa }k \left( {\frac{{x}^{ \beta }}{\beta }}+{\frac{ \left( \kappa +1 \right) l{k}^{2}{t}^{\alpha }}{\alpha }} \right) \right) {e}^{i \left( -{\frac{m{x}^{\beta }}{\beta } }+{\frac{\omega \,{t}^{\alpha }}{\alpha }}+\theta \right) }-c_{{1}} \sqrt{\kappa }{e}^{i \left( -{\frac{m{x}^{\beta }}{\beta }}+{\frac{ \omega \,{t}^{\alpha }}{\alpha }}+\theta \right) }\\&\quad \times \left( \tan \left( \sqrt{\kappa }k \left( {\frac{{x}^{\beta }}{\beta }}+{\frac{ \left( \kappa +1 \right) l{k}^{2}{t}^{\alpha }}{\alpha }} \right) \right) \right) ^{-1}. \end{aligned} \end{aligned}$$

or

30 $$\begin{aligned} \begin{aligned} F_9(x,t)&=c_{{1}}\sqrt{\kappa }\cot \left( \sqrt{\kappa }k \left( {\frac{{x}^{ \beta }}{\beta }}+{\frac{ \left( \kappa +1 \right) l{k}^{2}{t}^{\alpha }}{\alpha }} \right) \right) {e}^{i \left( -{\frac{m{x}^{\beta }}{\beta } }+{\frac{\omega \,{t}^{\alpha }}{\alpha }}+\theta \right) }+c_{{1}} \sqrt{\kappa }{e}^{i \left( -{\frac{m{x}^{\beta }}{\beta }}+{\frac{ \omega \,{t}^{\alpha }}{\alpha }}+\theta \right) }\\&\quad \times \left( \cot \left( \sqrt{\kappa }k \left( {\frac{{x}^{\beta }}{\beta }}+{\frac{ \left( \kappa +1 \right) l{k}^{2}{t}^{\alpha }}{\alpha }} \right) \right) \right) ^{-1}. \end{aligned} \end{aligned}$$

### Family 6

 When $\kappa =0$, then Eq. (1) have the following solitary wave solutions:

31 $$\begin{aligned} \begin{aligned} F_{10}(x,t)&=c_{{1}}{e}^{i \left( -{\frac{m{x}^{\beta }}{\beta }}+{\frac{\omega \,{t }^{\alpha }}{\alpha }}+\theta \right) }{k}^{-1} \left( {\frac{{x}^{ \beta }}{\beta }}+{\frac{ \left( \kappa +1 \right) l{k}^{2}{t}^{\alpha }}{\alpha }} \right) ^{-1}\\&\quad +c_{{1}}\kappa \,k \left( {\frac{{x}^{\beta }}{ \beta }}+{\frac{ \left( \kappa +1 \right) l{k}^{2}{t}^{\alpha }}{\alpha } } \right) {e}^{i \left( -{\frac{m{x}^{\beta }}{\beta }}+{\frac{\omega \,{t}^{\alpha }}{\alpha }}+\theta \right) }. \end{aligned} \end{aligned}$$

Assuming case 3, we get the following families of solutions:

### Family 7

When $\kappa <0$ then Eq. (1) have the following solitary wave solutions:

32 $$\begin{aligned} \begin{aligned} F_{11}(x,t)&=-1/2\,\sqrt{-{\kappa }^{-1}}c_{{0}}\sqrt{-\kappa }\tanh \left( \sqrt{ -\kappa }k \left( {\frac{{x}^{\beta }}{\beta }}+{\frac{ \left( 4\, \kappa +1 \right) l{k}^{2}{t}^{\alpha }}{\alpha }} \right) \right) {e}^{ i \left( -{\frac{m{x}^{\beta }}{\beta }}+{\frac{\omega \,{t}^{\alpha }}{ \alpha }}+\theta \right) }\\&\quad +c_{{0}}{e}^{i \left( -{\frac{m{x}^{\beta }}{ \beta }}+{\frac{\omega \,{t}^{\alpha }}{\alpha }}+\theta \right) }\\&\quad +1/2\, \sqrt{-{\kappa }^{-1}}c_{{0}}\kappa \,{e}^{i \left( -{\frac{m{x}^{ \beta }}{\beta }}+{\frac{\omega \,{t}^{\alpha }}{\alpha }}+\theta \right) }{\frac{1}{\sqrt{-\kappa }}} \left( \tanh \left( \sqrt{-\kappa }k \left( {\frac{{x}^{\beta }}{\beta }}+{\frac{ \left( 4\,\kappa +1 \right) l{k}^{2}{t}^{\alpha }}{\alpha }} \right) \right) \right) ^{-1 }. \end{aligned} \end{aligned}$$

or

**Figure 1:**
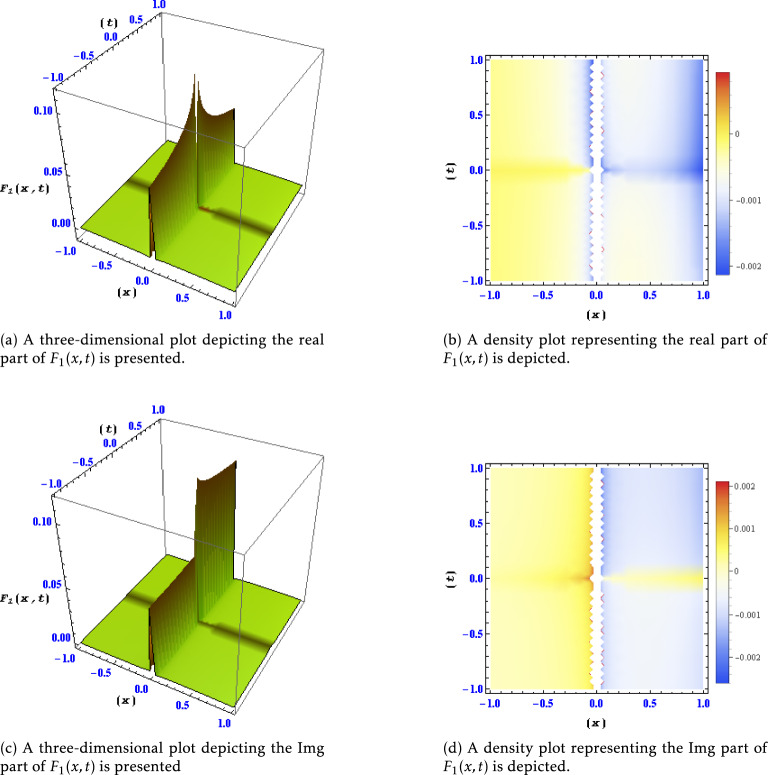
Different levels of detail are shown for the real and imaginary parts of the solution $F_1(x,t)$ in this plots.

**Figure 2:**
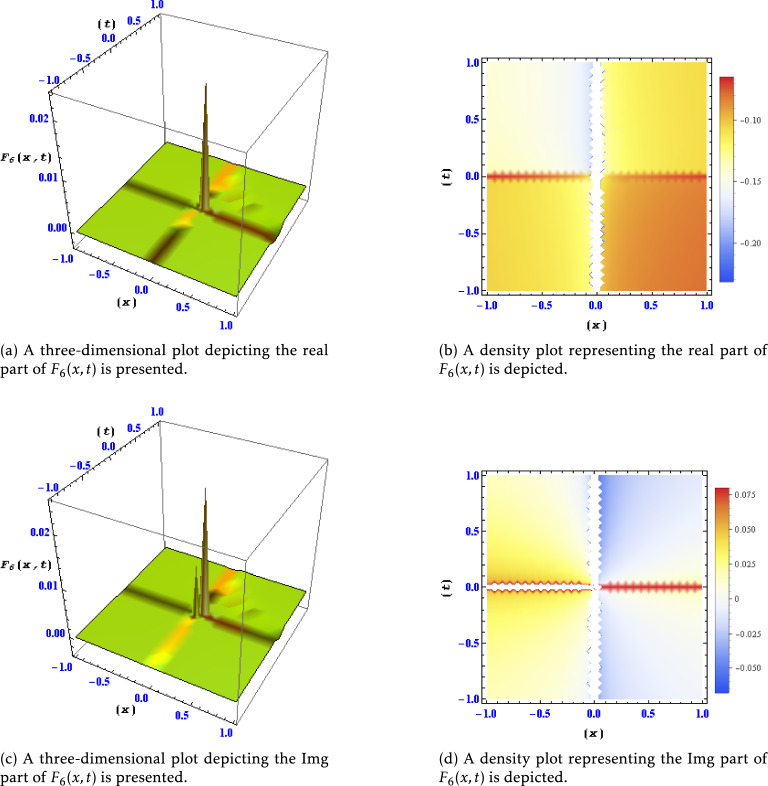
Different levels of detail are shown for the real and imaginary parts of the solution $F_6(x,t)$ in this plots.

**Figure 3:**
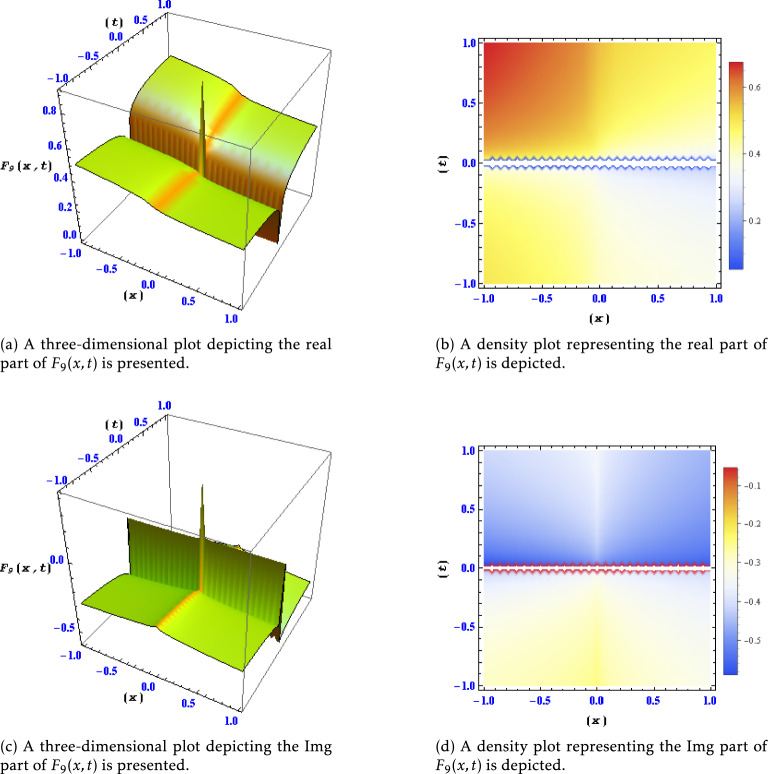
Different levels of detail are shown for the real and imaginary parts of the solution $F_{9}(x,t)$ in this plots.

**Figure 4:**
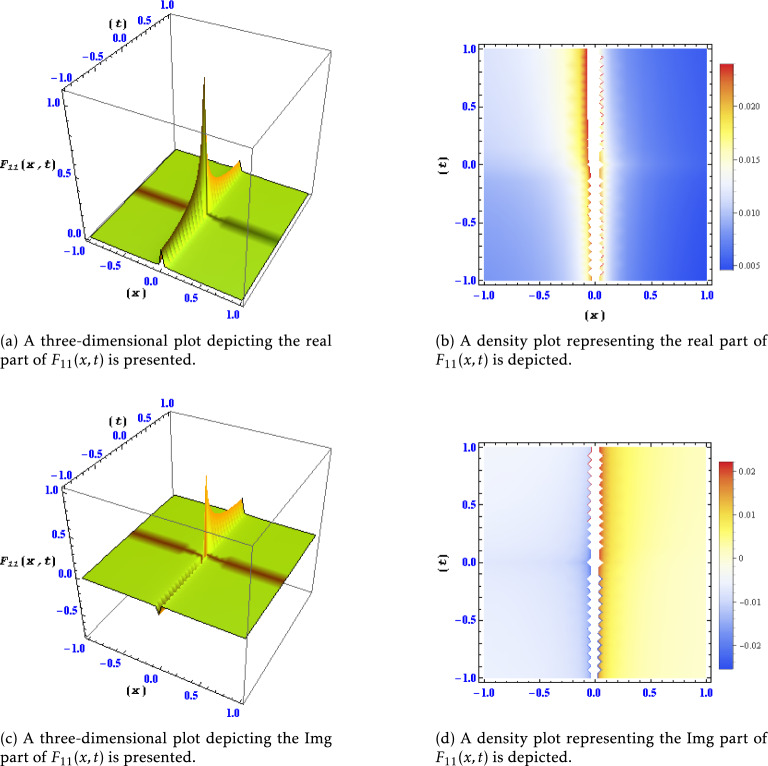
Different levels of detail are shown for the real and imaginary parts of the solution $F_{11}(x,t)$ in this plots.

**Figure 5:**
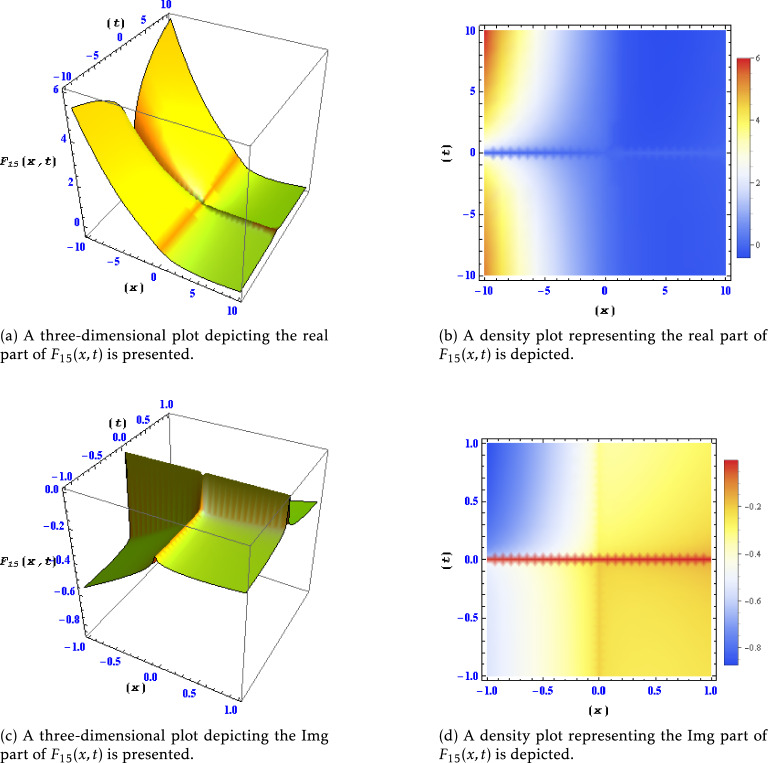
Different levels of detail are shown for the real and imaginary parts of the solution $F_{15}(x,t)$ in this plots.

33 $$\begin{aligned} \begin{aligned} F_{12}(x,t)&=-1/2\,\sqrt{-{\kappa }^{-1}}c_{{0}}\sqrt{-\kappa }\coth \left( \sqrt{ -\kappa }k \left( {\frac{{x}^{\beta }}{\beta }}+{\frac{ \left( 4\, \kappa +1 \right) l{k}^{2}{t}^{\alpha }}{\alpha }} \right) \right) {e}^{ i \left( -{\frac{m{x}^{\beta }}{\beta }}+{\frac{\omega \,{t}^{\alpha }}{ \alpha }}+\theta \right) }\\&\quad +c_{{0}}{e}^{i \left( -{\frac{m{x}^{\beta }}{ \beta }}+{\frac{\omega \,{t}^{\alpha }}{\alpha }}+\theta \right) }\\&\quad +1/2\, \sqrt{-{\kappa }^{-1}}c_{{0}}\kappa \,{e}^{i \left( -{\frac{m{x}^{ \beta }}{\beta }}+{\frac{\omega \,{t}^{\alpha }}{\alpha }}+\theta \right) }{\frac{1}{\sqrt{-\kappa }}} \left( \coth \left( \sqrt{-\kappa }k \left( {\frac{{x}^{\beta }}{\beta }}+{\frac{ \left( 4\,\kappa +1 \right) l{k}^{2}{t}^{\alpha }}{\alpha }} \right) \right) \right) ^{-1 }. \end{aligned} \end{aligned}$$

### Family 8

 When $\kappa >0$ then Eq. (1) have the following solitary wave solutions:

34 $$\begin{aligned} \begin{aligned} F_{13}(x,t)&=1/2\,\sqrt{-{\kappa }^{-1}}c_{{0}}\sqrt{\kappa }\tan \left( \sqrt{ \kappa }k \left( {\frac{{x}^{\beta }}{\beta }}+{\frac{ \left( 4\,\kappa +1 \right) l{k}^{2}{t}^{\alpha }}{\alpha }} \right) \right) {e}^{i \left( -{\frac{m{x}^{\beta }}{\beta }}+{\frac{\omega \,{t}^{\alpha }}{ \alpha }}+\theta \right) }+c_{{0}}{e}^{i \left( -{\frac{m{x}^{\beta }}{ \beta }}+{\frac{\omega \,{t}^{\alpha }}{\alpha }}+\theta \right) }\\&\quad -1/2\, \sqrt{-{\kappa }^{-1}}c_{{0}}\sqrt{\kappa }{e}^{i \left( -{\frac{m{x} ^{\beta }}{\beta }}+{\frac{\omega \,{t}^{\alpha }}{\alpha }}+\theta \right) } \left( \tan \left( \sqrt{\kappa }k \left( {\frac{{x}^{ \beta }}{\beta }}+{\frac{ \left( 4\,\kappa +1 \right) l{k}^{2}{t}^{ \alpha }}{\alpha }} \right) \right) \right) ^{-1}. \end{aligned} \end{aligned}$$

or

35 $$\begin{aligned} \begin{aligned} F_{14}(x,t)&=-1/2\,\sqrt{-{\kappa }^{-1}}c_{{0}}\sqrt{\kappa }\cot \left( \sqrt{ \kappa }k \left( {\frac{{x}^{\beta }}{\beta }}+{\frac{ \left( 4\,\kappa +1 \right) l{k}^{2}{t}^{\alpha }}{\alpha }} \right) \right) {e}^{i \left( -{\frac{m{x}^{\beta }}{\beta }}+{\frac{\omega \,{t}^{\alpha }}{ \alpha }}+\theta \right) }+c_{{0}}{e}^{i \left( -{\frac{m{x}^{\beta }}{ \beta }}+{\frac{\omega \,{t}^{\alpha }}{\alpha }}+\theta \right) }\\&\quad +1/2\, \sqrt{-{\kappa }^{-1}}c_{{0}}\sqrt{\kappa }{e}^{i \left( -{\frac{m{x} ^{\beta }}{\beta }}+{\frac{\omega \,{t}^{\alpha }}{\alpha }}+\theta \right) } \left( \cot \left( \sqrt{\kappa }k \left( {\frac{{x}^{ \beta }}{\beta }}+{\frac{ \left( 4\,\kappa +1 \right) l{k}^{2}{t}^{ \alpha }}{\alpha }} \right) \right) \right) ^{-1}. \end{aligned} \end{aligned}$$

### Family 9

When $\kappa =0$, then Eq. (1) have the following solitary wave solutions:

36 $$\begin{aligned} \begin{aligned} F_{15}(x,t)&=-1/2\,\sqrt{-{\kappa }^{-1}}c_{{0}}{e}^{i \left( -{\frac{m{x}^{\beta } }{\beta }}+{\frac{\omega \,{t}^{\alpha }}{\alpha }}+\theta \right) }{k}^{ -1} \left( {\frac{{x}^{\beta }}{\beta }}+{\frac{ \left( 4\,\kappa +1 \right) l{k}^{2}{t}^{\alpha }}{\alpha }} \right) ^{-1}+c_{{0}}{e}^{i \left( -{\frac{m{x}^{\beta }}{\beta }}+{\frac{\omega \,{t}^{\alpha }}{ \alpha }}+\theta \right) }\\&\quad +1/2\,\sqrt{-{\kappa }^{-1}}c_{{0}}\kappa \,k \left( {\frac{{x}^{\beta }}{\beta }}+{\frac{ \left( 4\,\kappa +1 \right) l{k}^{2}{t}^{\alpha }}{\alpha }} \right) {e}^{i \left( -{\frac{m{x}^{\beta }}{\beta }}+{\frac{\omega \,{t}^{\alpha }}{\alpha }}+\theta \right) }. \end{aligned} \end{aligned}$$

## Results and discussion

This work introduces the use of the Backlund transformation along with the Riccati–Bernoulli sub-ODE technique, to enhance the comprehension of the frictional order Kundu–Eckhaus equation among the scientific community. By doing this, we discover a wide range of solution categories that have not been studied before. By utilising the Backlund transformation, our method produces greatly improved solutions in comparison to traditional analytical techniques, which frequently fail to encompass the complete range of physical events. Our new technique eliminates the requirement for linearization procedures widely used in existing literature, providing accurate solutions to nonlinear issues in the field of physical sciences. The Backlund transformation, used in this research, has demonstrated its effectiveness in addressing different physical phenomena, highlighting its efficacy.

Our unique fractional-order solutions offer a significant departure from conventional integer-order answers, which frequently fall short in fully encompassing the entire range of physical events. Our method effectively obtains correct solitary wave solutions, including powerful peaks and dark valley-shaped waves, contributing to a deeper understanding of complex physical phenomena. They are particularly useful for modelling solitons in optical fibres, analysing shock waves in fluid mechanics, and comprehending solitons in plasma physics. In order to illustrate the characteristics of some periodic and solitary solutions, we utilise the Mathematica software and specify specific parameter values within the exact solutions. The succeeding figures visually describe the properties of the solutions, offering a clear and physical depiction of the results.

Figure 1: Real and Imaginary Parts of Solution $F_1(x,t)$ at Different Detail Levels:

This figure exhibits various levels of detail for both the real and imaginary components of the solution $F_1(x,t)$. The different plots illustrate how the solution evolves and changes concerning different spatial and temporal resolutions, offering insights into the intricate behavior of $F_1(x,t)$. Figure 2: Real and Imaginary Parts of Solution $F_6(x,t)$ at Different Detail Levels:

Similar to Fig. 1, this figure showcases different levels of detail for the real and imaginary parts of the solution $F_6(x,t)$. The plotted variations enable a detailed examination of the behavior and characteristics of $F_6(x,t)$ under different resolutions. Figure 3: Real and Imaginary Parts of Solution $F_{9}(x,t)$ at Different Detail Levels:

This figure portrays the real and imaginary components of the solution $F_{9}(x,t)$ across varying levels of detail. The plotted data allows for a comprehensive understanding of the features and fluctuations present in $F_{9}(x,t)$ under different spatial and temporal resolutions. Figure 4: Real and Imaginary Parts of Solution $F_{11}(x,t)$ at Different Detail Levels:

Similarly, this figure illustrates the behavior of the real and imaginary parts of the solution $F_{11}(x,t)$ at different levels of detail. It provides an in-depth analysis of how $F_{11}(x,t)$ changes and evolves concerning varying spatial and temporal resolutions. Figure 5: Real and Imaginary Parts of Solution $F_{15}(x,t)$ at Different Detail Levels:

This figure exhibits the real and imaginary components of the solution $F_{15}(x,t)$ across different levels of detail. It demonstrates the intricate details and variations within $F_{15}(x,t)$ under different spatial and temporal scales, aiding in a comprehensive understanding of its behavior.

## Conclusion

This work presents a new method that uses the Riccati–Bernoulli sub-ODE technique to provide accurate solutions for important fractional-order nonlinear partial differential equations related to soliton waves. In order to accomplish this, we utilise the backlund transformation approach. More precisely, we utilise this approach to derive novel precise solutions for the frictional order Kundu–Eckhaus equation (FKEe). We utilise symbolic computation technologies like Maple and Mathematica to compute and verify these new solutions for the fractional equations under consideration. These solutions have the potential to significantly improve our comprehension of the physical consequences of the underlying nonlinear fractional model. Our investigation of fractional-order systems is a significant divergence from conventional integer-order techniques, revealing multiple important benefits. Fractional-order systems provide a more complicated and adaptable modelling framework than integer-order systems, which may have trouble encapsulating the entire intricacy and dynamics of real-world events. Fractional-order dynamics is especially useful for modelling processes with complex dynamics or anomalous behaviours that are outside the scope of traditional approaches. It allows for a more realistic portrayal of systems with non-integer behaviours. Moreover, the effectiveness of our suggested method is demonstrated by the extraction of a wide range of solutions, highlighting its adaptability to real-world modelling. Because of its adaptability, our model may be customised to fit many elements of the observed events, which is crucial for tackling the complexity and variety that are frequently encountered in actual applications. The potential to find various answers is a useful tool that gives researchers and practitioners options and improves our ability to fully comprehend complex and multifaceted systems. This approach can be used to various fractional-order nonlinear partial differential equations, provided that the homogeneous balancing assumption is satisfied. In addition, we thoroughly verify that all acquired solutions meet the original fractional-order nonlinear partial differential equations to guarantee the accuracy of our conclusions. The answers we have derived make a significant contribution by providing fresh and precise soliton wave solutions for space-time fractional-order nonlinear partial differential equations in the scholarly literature.

## Data Availability

The data sets used and/or analysed during the current study available from the corresponding author on reasonable request.
